# Effects of Climate Change on the Distribution of Threatened Fishing Bat *Myotis pilosus* in China

**DOI:** 10.3390/ani13111784

**Published:** 2023-05-27

**Authors:** Wei Guo, Zixuan Li, Tong Liu, Jiang Feng

**Affiliations:** 1 College of Life Science, Jilin Agricultural University, Changchun 130118, China; 2 Jilin Provincial Key Laboratory of Animal Resource Conservation and Utilization, Northeast Normal University, Changchun 130117, China; 3 Key Laboratory of Vegetation Ecology of Education Ministry, Institute of Grassland Science, Northeast Normal University, Changchun 130024, China

**Keywords:** climate change, MaxEnt, suitable habitat, climate refugia, *Myotis pilosus*

## Abstract

**Simple Summary:**

*Myotis pilosus* is a globally “Vulnerable” species. As the only known fishing bat in East Asia, *M. pilosus* is mainly distributed in China, and the protection of Chinese *M. pilosus* is of great significance for its persistence. Therefore, we collected species distribution data of *M. pilosus* from China and applied MaxEnt to assess its habitat suitability, recognize the important environmental variables, predict future distribution changes, and identify the potential future climate refugia. The results showed that temperature and precipitation, especially the minimum temperature, could be important environmental factors affecting the distribution of *M. pilosus*. The current suitable habitats of *M. pilosus* are located primarily in southwest and southeast China. Under future climate scenarios, the suitable habitats were expected to expand and shift toward higher latitudes and altitudes, but the area of predicted suitable habitats that *M. pilosus* could disperse and successfully colonize will be reduced in 2050 and 2070. Thus, five regions were identified as potential future climate refugia and suggested to be under priority protection and long-term monitoring. This study provides helpful information on the possible distribution changes of *M. pilosus* under current and future climate scenarios, which is important for the conservation of this vulnerable piscivorous bat.

**Abstract:**

Climate change and biodiversity loss are two severe challenges that the world is facing. Studying the distribution shifts of species in response to climate change could provide insights into long-term conservation and biodiversity maintenance. *Myotis pilosus* is the only known fishing bat in East Asia, whereas its population has been decreasing in recent years and it is listed as a “Vulnerable” species. To assess the impact of climate change on the distribution of *M. pilosus*, we obtained 33 *M. pilosus* occurrence records within China where they are mainly distributed, and extracted 30 environmental variables. MaxEnt was applied to assess the habitat suitability, recognize the important environmental variables, predict future distribution changes, and identify the potential future climate refugia. The prediction result based on eleven dominant environmental variables was excellent. The Jackknife test showed that the “minimum temperature of coldest month”, “precipitation of wettest quarter”, “percent tree cover”, and “precipitation of driest month” were the main factors affecting the distribution of *M. pilosus*. The current suitable areas were predicted to be mainly located in southwest and southeast China with a total area of about 160.54 × 10^4^ km^2^, accounting for 16.72% of China’s land area. Based on the CCSM4, it was predicted that the future (2050 and 2070) suitable areas of *M. pilosus* will expand and shift to high latitudes and altitudes with global warming, but the area of moderately and highly suitable habitats will be small. Considering the dispersal capacity of *M. pilosus*, the area of colonized suitable habitats in 2050 and 2070 was predicted to be only ca. 94 × 10^4^ km^2^ and 155 × 10^4^ km^2^, respectively. The central and southern parts of Hainan, southern Guangdong, central Guizhou, and southern Beijing were identified as potential climate refugia and could be considered as priority conservation areas for *M. pilosus*. Thus, we suggest long-term monitoring of the priority conservation areas, especially the areas at high latitudes and altitudes. These results contribute to our knowledge of the possible spatial distribution pattern of *M. pilosus* under current and future climate scenarios, which is important for the population protection and habitat management of this special piscivorous bat species.

## 1. Introduction

Two severe challenges that the world is facing are climate change and biodiversity loss [1]. The Living Planet Report 2022, published by the World Wide Fund for Nature, showed that the relative abundance of global wildlife populations declined by an average of 69% between 1970 and 2018 [2]. Climate change is likely to be the leading cause of species loss or extinction over the past century [3]. With the accelerating rate of species extinction, the conservation of biodiversity has become the focus of biological research [4].

Bats are one of the most sensitive mammals to climate change [5,6]. As the only mammals with true powered flight, bats are widely distributed, have extremely high species diversity, and play important roles in ecosystems. However, because of the slow reproductive rate, high metabolism and longevity, as well as habitat specialization, bats are particularly sensitive to the effects of climate change [7,8,9]. According to the study of *Tadarida teniotis*, years of drought largely impaired the reproduction of *T. teniotis* in the Mediterranean region and led to a significant reduction in pregnant or lactating females and juveniles [10]. A similar situation was reported by a study of vespertilionid bats in western North America; when the temperature was greater than average and the precipitation was less than average, the reproductive output of bat populations reduced [11]. The study of *Myotis daubentonii* found that the increased rainfall could also lead to reduced reproductive success, especially when extreme weather events (such as excessive summer rainfall) occur [12]. These studies indicated that climate change affects bat populations, while the specific impacts may vary by species and environmental factors. To date, over 16% of bats are listed as threatened species in the IUCN Red List [13], and more than half of the bat populations have declined dramatically due to climate change, habitat degradation, habitat destruction, etc. [14]. 

Rickett’s big-footed bat, *Myotis pilosus*, is mainly distributed in China and very scattered in Laos and Vietnam [13]. This species is the only known fishing bat in East Asia [15], preying on both insects and freshwater fishes [16,17,18,19], and has a relatively large body size with well-developed feet and claws [16]. *Myotis pilosus* generally inhabits forested hills, mountains, and wetlands [19,20,21], and forages from the water surface of nearby reservoirs, ponds, and rivers [17,22,23]. In addition, hibernation was observed in the Beijing population of this species from November to mid-March of the following year [17]. Many researchers focused on this species and made significant progress in understanding the genetic basis of echolocation system specialization [24], the role of paleoclimate in shaping the current population genetic structure [25], the reason for dietary niche expansion from insects to fish [18], and the evolution of taste receptor genes [26]. However, according to the IUCN Red List, *M. pilosus* is globally “Vulnerable” and its population is declining year by year [19]. For species conservation and ongoing research, there is an urgent need to assess the survival potential and habitat suitability of *M. pilosus*.

Species distribution model (SDM), also known as ecological niche model, is a mathematical model based on species existence records and environmental data [27]. SDM associates the information of species occurrence records and the environmental characteristics on the occurrence sites, and infers the relationship between species occurrence and environmental data. Based on the relationship, the SDM can further predict the potential distribution areas that meet the ecological requirements of the species [28,29]. SDMs play an important role in conservation biology research [30] and have been widely used to study species’ responses to climate change [31,32], potential distribution area predictions [33], impacts of local climate change on species richness and community stability [34,35], protected area delineation for endangered species, and impacts of human activities on endangered species [36,37]. Among the different species distribution models, maximum entropy modeling (MaxEnt) is widely used in the study of species suitable habitat assessment because of its high prediction accuracy and stability [38,39,40,41].

In this study, we collected the distribution data of *M. pilosus* in China and predicted the potentially suitable habitats using MaxEnt. The aims of this study were: (1) to recognize important environmental variables that influence the distribution of *M. pilosus*; (2) to predict the spatial distribution pattern of *M. pilosus* under current and future climate scenarios; and (3) to identify the potential future climate refugia. Our results will demonstrate the likely distribution of *M. pilosus* under current and future climate scenarios, help to understand the effects of environmental factors on the distribution and survival of this species, and provide fundamental data for the protection and management of this ecologically important species.

## 2. Materials and Methods

### 2.1. Species Distribution Data

**Figure 1:**
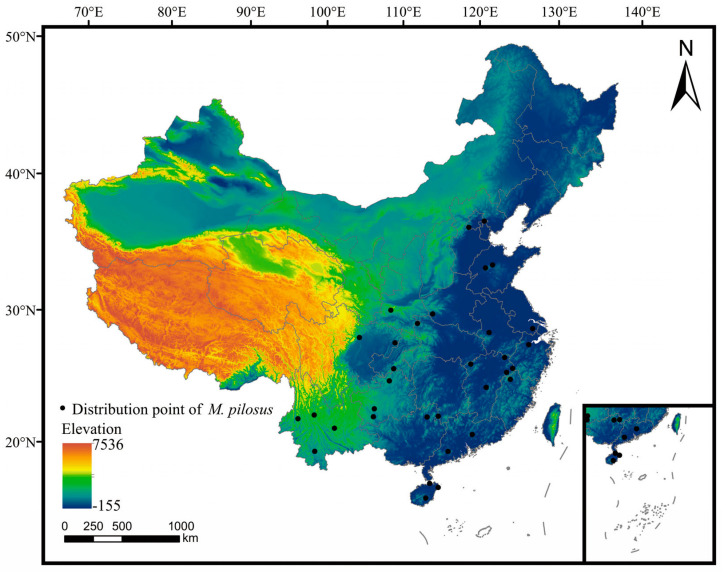
The distribution points of *M. pilosus* in China. The map was based on the standard map No. GS (2019) 1822 downloaded from the Standard Mapping Service website of the Natural Resource of the People’s Republic of China (http://bzdt.ch.mnr.gov.cn/, accessed on 10 October 2022). The base map has no modifications, and the geographical coordinate is WGS-84.

The distribution data of *M. pilosus* was collected from the Global Biodiversity Information Agency (https://www.gbif.org/, accessed on 10 October 2022), scientific literature, and field survey data (2006–2022). The distribution points were loaded into ArcGIS v.10.4, and ENMTools was used to eliminate the autocorrelation and ensure that there was only one distribution point in each 2.5 min grid cell (about 4.5 km × 4.5 km). Finally, a total of 33 distribution points were kept for subsequent analyses (Figure 1; Table S1).

### 2.2. Environmental Variables

A total of 30 environmental variables were collected and divided into six categories: climate, land cover and vegetation, terrain, light index, human disturbance, and river (Table 1). Nineteen climate variables were downloaded from the WorldClim database (http://www.worldclim.org, accessed on 10 October 2022). Percent tree cover and normalized vegetation index were downloaded from the Earth Science Data and Information System Engineering website (https://earthdata.nasa.gov/, accessed on 10 October 2022). Land use, light index, settlements, roads, and river data were obtained from the Resource and Environmental Science and Data Center of the Chinese Academy of Sciences (https://www.resdc.cn/, accessed on 26 April 2023). Three terrain variables were extracted from DEM digital elevation data, which was obtained from the Computer Network Information Center of the Chinese Academy of Sciences and the International Scientific Data Website (http://www.gscloud.cn/, accessed on 10 October 2022). The human influence index data was downloaded from Socioeconomic Data and Applications Center (https://sedac.ciesin.columbia.edu/, accessed on 28 April 2023). Based on the CCSM4 which has a high accuracy for climate simulation and prediction [42], future (2050 and 2070) climate projections were reconstructed under three representative concentration pathway (RCP) scenarios, i.e., RCP2.6, RCP4.5, and RCP8.5, with increasing atmospheric concentrations of carbon dioxide and other greenhouse gases and aerosols [43]. The spatial resolution of all environmental variables was adjusted to 2.5 min by ArcGIS, and the environmental variables were cut by the Chinese administrative map downloaded from the Standard Mapping Service website of the Natural Resource of the People’s Republic of China (GS (2019) 1822, http://bzdt.ch.mnr.gov.cn/, accessed on 10 October 2022). All the maps in this study represent the authors’ views and are not used for political purposes.

The distribution data and environmental variables were imported into MaxEnt v.3.4.4 for simulation. All the simulations were repeated ten times, and the environmental variables with a contribution rate of less than 1% were eliminated (Table S2). High spatial correlations between environmental variables will reduce the accuracy of MaxEnt. Thus, ENMTools was used to examine the correlations between environmental variables. For the environmental variables with an absolute value of correlation coefficient greater than 0.75, only those with higher contribution rates were retained (Figure S1). Finally, eleven environmental variables (Aspect, Bio6, Bio14, Bio15, Bio16, Dis_riv, Dis_set, Landuse, Light, Slope, and TREE) were kept for subsequent model reconstruction.

### 2.3. MaxEnt Procedures

Distribution data and eleven environmental variables were imported into MaxEnt. Seventy-five percent of the distribution data was used for model training, and the remaining 25% was used for testing. The model was repeated ten times, and the replicate runs were subsampled for model accuracy. The model results were outputted in logistic format.

The Jackknife test was used to evaluate the importance of each environmental variable. The influence of the environmental variable on the distribution was visualized by an environmental variable response curve. The accuracy of the model prediction was measured using AUC (the area value under the receiver operating characteristic curve). The value of AUC ranged from zero to one. The higher the AUC, the better the model’s performance, with the prediction considered failed (AUC 0.5–0.6), poor (0.6–0.7), fair (0.7–0.8), good (0.8–0.9), or excellent (0.9–1) [44].

### 2.4. Classification of Suitable Habitats

Habitat suitability is typically represented by a value from zero to one, where the higher the value is, the greater probability of occurrence (*p*). According to the maximum test sensitivity plus specificity threshold (MTSPS), the prediction results were divided into suitable habitat and unsuitable habitat [45]. There were four grades of suitability: unsuitable habitat (*p* < MTSPS), lowly suitable habitat (MTSPS < *p* < 0.5), moderately suitable habitat (0.5 < *p* < 0.7), and highly suitable habitat (0.7 < *p* < 1). The SDM Toolbox tool was used to calculate the changes in centroid positions under different scenarios.

### 2.5. Dispersal Simulations

We used the “MigClim” package [46] in R v.4.2.3 [47] to simulate the dispersal of *M. pilosus* and incorporate species dispersal limits into the species distribution predictions. The species’ initial distribution points and habitat suitability maps in 2050 and 2070 generated from the MaxEnt models were used as input files. The reclassification threshold was set to 208 according to the MTSPS, and environmental change step number was set to 2. We assumed that our species can disperse once a year, so the dispersal step number was adjusted to 20. According to Ma et al. [17], *M. pilosus* can forage about 8 km away from its roosts, almost twice that of the grid cell used in this analysis, so we modified the dispersal kernel to c(1.0, 1.0, 0.4, 0.16, 0.06). We defined the maximum long-distance dispersal distance as 15 km based on the research of congenus species, *Myotis bechsteinii* and *Myotis emarginatus* [48]. The remaining parameters were set using the default values.

### 2.6. Identification of Climate Refugia

A conservative prediction of future climate refugia was obtained by merging the maps of highly suitable habitats under current and future climate scenarios. In this study, two types of refugia were identified [49,50,51]: (1) in situ refugia, which are the most important areas for species and highly suitable under all climate scenarios, (2) ex situ refugia, which are un-highly suitable areas for the species under current climate conditions, but highly suitable under all future scenarios. Although in situ refugia are crucial for the survival and reproduction of species, ex situ refugia are important for promoting resilience in the future. The intersection of climate refugia under three climate scenarios was obtained in 2050 and 2070 and projected to the range of *M. pilosus*, which was downloaded from the IUCN Red List (https://www.iucnredlist.org/, accessed on 10 October 2022) to determine the priority conservation areas.

## 3. Results

### 3.1. Model Verification and Environmental Variables

**Figure 2:**
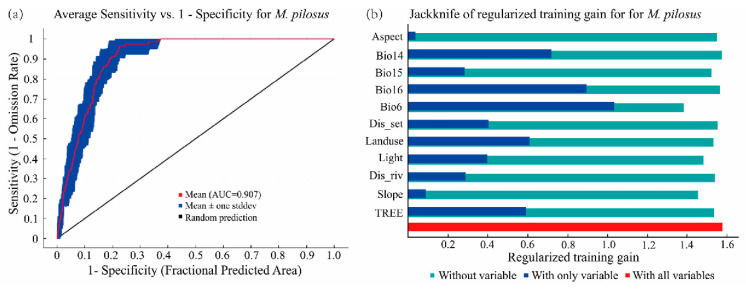
(**a**) Receiver operating characteristic (ROC) curve and AUC values for MaxEnt results averaged over ten replicate runs; (**b**) Jackknife test of the importance of eleven dominant environmental variables used in the *M. pilosus* potential distribution modeling.

The average AUC value of ten replications was higher than 0.9 (Figure 2a), indicating that MaxEnt had an excellent predictive ability and was able to accurately predict the potential distribution of *M. pilosus*. According to the results of the Jackknife test (Figure 2b), minimum temperature of coldest month (Bio6) contributed most to the distribution of *M. pilosus*, followed by precipitation of wettest quarter (Bio16), percent tree cover (TREE), and precipitation of driest month (Bio14), with the cumulative contribution totaling up to 72.4% (Table 2).

**Figure 3:**
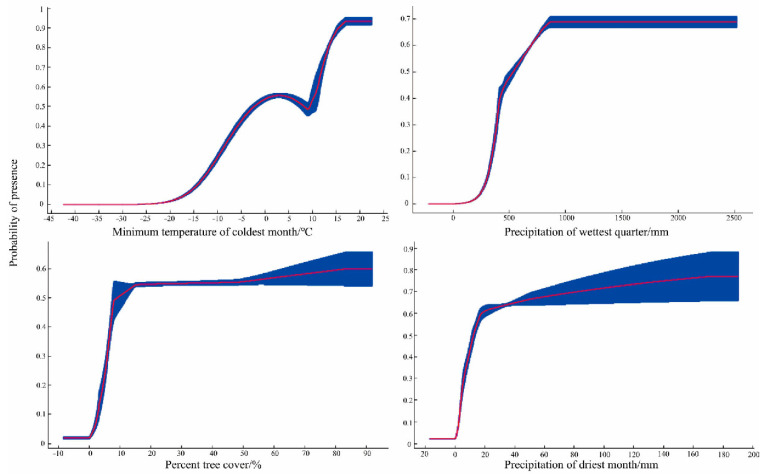
Response curve of the four most important environmental variables. The *x*-axis indicates the environmental variable, the *y*-axis shows the probability of presence, the red curve represents the mean response, and the blue band represents the standard deviation (±SD) calculated from ten replicates.

The response curves of these four environmental variables show a marked increase in the occurrence probability with increasing levels of temperature, precipitation, and tree cover (Figure 3). According to the response curves and MTSPS threshold (0.2806), *M. pilosus* prefers habitats with warm temperatures (Bio6 > −8.9 °C), humid climates (Bio14 > 5.33 mm and Bio16 > 384.48 mm), and high percent tree cover (TREE > 5.68%).

### 3.2. Current Potential Suitable Habitats

**Figure 4:**
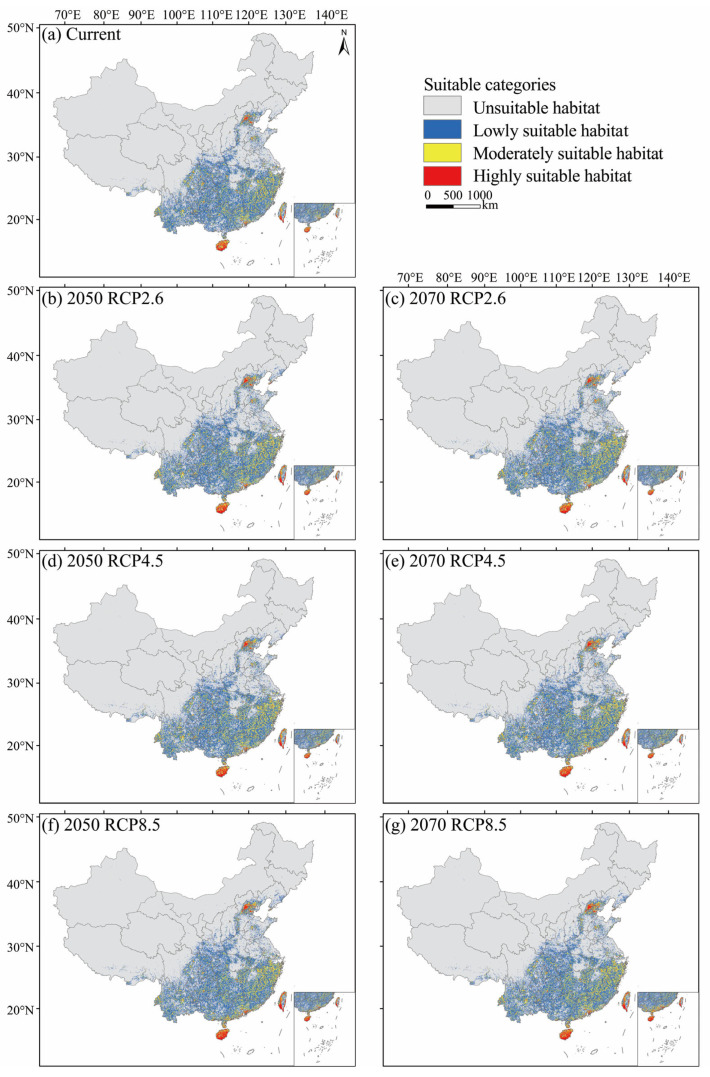
Potential suitable habitats of *M. pilosus* in different years and under different climate scenarios. The potential distribution of *M. pilosus* in (**a**) current, (**b**) 2050 RCP2.6, (**c**) 2070 RCP2.6, (**d**) 2050 RCP4.5, (**e**) 2070 RCP4.5, (**f**) 2050 RCP8.5, and (**g**) 2070 RCP8.5 conditions.

Under the current climatic condition, the suitable habitats of *M. pilosus* were predicted to be mainly distributed in southwest and southeast China (Figure 4a and Figure S2), at an average altitude of 648.19 m, and covered a total area of 160.54 × 10^4^ km^2^ accounting for 16.72% of China’s land area (Table 3). The habitats with high, moderate, and low suitability were predicted to be 5.74 × 10^4^ km^2^, 34.82 × 10^4^ km^2^, and 119.98 × 10^4^ km^2^, respectively (Table 3). The moderately and highly suitable habitats were predicted to be mainly distributed in southern coastal areas and Beijing (Figure 4a).

### 3.3. Future Potential Suitable Habitats

Compared with the current distribution, the average altitude and area of suitable habitats estimated under the unlimited dispersal assumption were both increased in the future climate scenarios (Table 3). The most obvious changes were observed under the RCP8.5 scenario in 2070, with an average altitude increase of 60.37 m and the total area of suitable habitats expanded by 18.46 × 10^4^ km^2^ (Figure 4; Table 3). More specifically, the areas of highly and moderately suitable habitats increased to 9.70 × 10^4^ km^2^ and 41.43 × 10^4^ km^2^, and the area of habitats with low suitability markedly expanded with an increase of 6.58% compared with the current value (Table 3).

**Figure 5:**
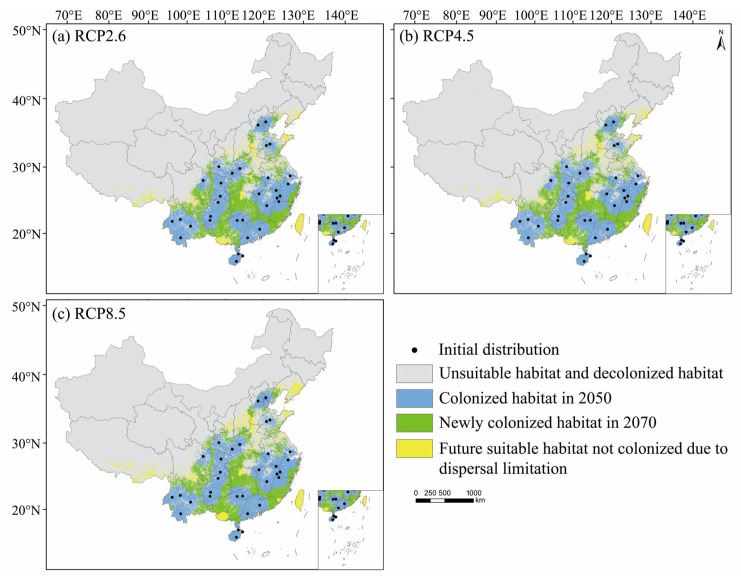
Dispersal simulation of *M. pilosus* under three climate scenarios (**a**) RCP2.6, (**b**) RCP4.5, (**c**) RCP8.5.

However, when considering the dispersal capacity of *M. pilosus*, the area of colonized suitable habitats in the future was decreased (Table 3). In 2050, the average area of colonized suitable habitats under three RCPs was 94.38 × 10^4^ km^2^ and accounted for 58.78% of the current suitable habitat area. In 2070, the area of colonized suitable habitats increased the most to 156.99 × 10^4^ km^2^ under the RCP8.5 scenario and reduced by 3.55 × 10^4^ km^2^ compared to the current suitable habitats. *Myotis pilosus* could disperse to most areas of southern China, and the northernmost regions could spread to the north of Heibei Province in 2050 and the center of Liaoning Province in 2070 (Figure 5).

### 3.4. Shift in Centroid Position

**Figure 6:**
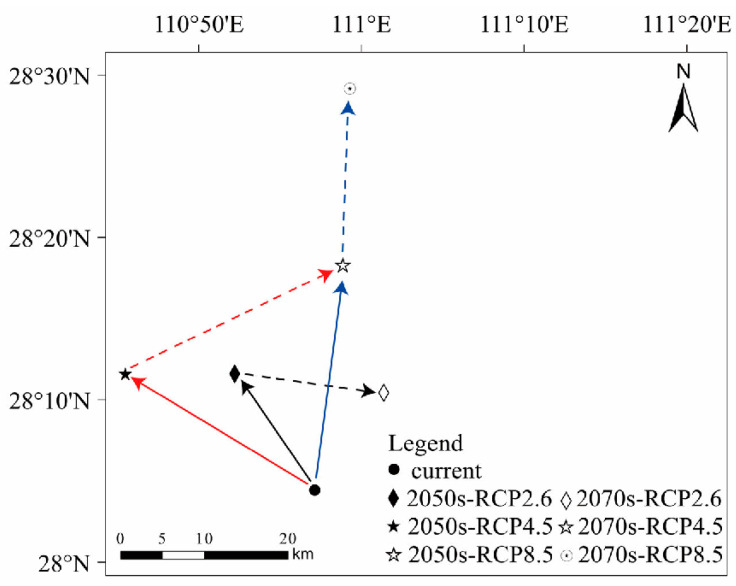
Centroid shifts under different climate scenarios for *M. pilosus*. The arrow indicates the direction of the predicted shift over time. Solid lines and dashed lines represent the shifts happen during current–2050 and 2050–2070, respectively. Different colors represent the different climate scenarios.

The current centroid of *M. pilosus* is located in Anhua County of Hunan Province. In the future climatic context, the centroid position was predicted to slightly shift to higher latitudes but was still located in the same county (Figure 6). Under the RCP2.6 climate scenario, the center of the potential suitable habitat shifted to the northwest by 15.50 km in 2050, and to the southeast by 15.14 km in 2070. Under the RCP4.5 climate scenario, the centroid position shifted to the northwest by 23.18 km in 2050, and to the northeast by 25.11 km in 2070. Under the RCP8.5 climate scenario, the centroid position shifted to the northeast by 25.83 km in 2050, and to the northeast by 20.13 km in 2070.

### 3.5. Future Climate Refugia

The area of in situ refugia was slightly reduced with the increase in greenhouse gas concentrations in 2050 and 2070 (Figure S3; Table 4). The area of ex situ refugia markedly increased under each scenario; the area under the RCP8.5 scenario was more than one and a half times that of RCP2.6 in 2050, and almost three times that in 2070 (Figure S3; Table 4).

**Figure 7:**
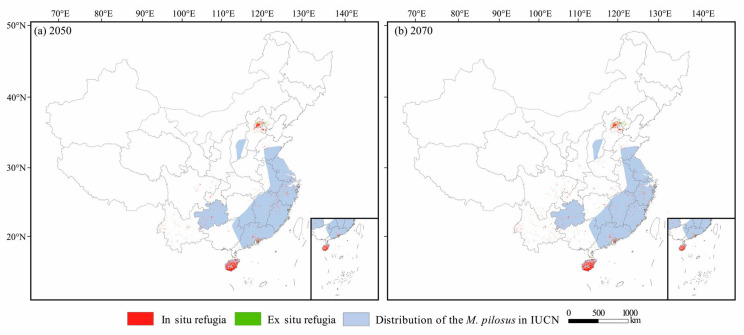
Distribution of in situ refugia (red) and ex situ refugia (green) in (**a**) 2050 and (**b**) 2070. Light blue areas represent the distribution of *M. pilosus* downloaded from IUCN Red List.

Several large patches of climate refugia were identified as priority conservation areas, including the central and southern Hainan Island, southern Guangdong Province, central Guizhou Province, and southern Beijing (Figure 7).

## 4. Discussion

Understanding the potential distribution of threatened species under current and future climate scenarios is critical to biological conservation and strategic planning formulation [52,53]. Occurrence data of threatened species are often insufficient, which hinders the design of appropriate conservation strategies [54]; thus, the species distribution model has been widely applied to fill the gap of insufficient data in species conservation. In this study, MaxEnt was used to predict the potential distribution of *M. pilosus* based on the data of 33 occurrence records within China. Although the data set of distribution is small, our simulation shows a high accuracy (Figure 2a). Similarly, Jiang et al. (2020) used MaxEnt to predict the suitable habitat of *Stachyris nonggangensis* with 33 occurrence records with a model accuracy of 0.99 [55], indicating that the model performed well with limited data. Silva et al. (2014) used only 17, 25, and 6 occurrence points to estimate the potential distribution of endemic lizard species *Tropidurus montanus*, *Cercosaura albostrigata*, and *Bachia oxyrhina* in the Brazilian Cerrado hotspot; the model accuracies were 0.99, 0.94, and 0.99, respectively [56], also suggesting that MaxEnt can obtain accurate simulation results with small data sets [57,58].

Bats are sensitive to changes in temperature and precipitation, as evidenced by the studies of *Pipistrellus kuhlii* in Europe [59] and 17 bat species in China over the past 50 years [60]. Similarly, in this study, we also found that the distribution of *M. pilosus* was mainly associated with four main environmental variables, including minimum temperature, percent tree cover, and minimum and maximum precipitation. Moreover, the minimum temperature was found to be the most important environmental variable (Figure 2b; Table 2), consistent with the study of *Hypsugo savii*, *Pipistrellus pipistrellus*, and *P. kuhlii*, suggesting that the lowest temperature may be an important environmental factor affecting the distribution of bats [59,61]. 

Temperature changes may affect the food resource availability, duration and intensity of hibernation, and thermal requirements during reproduction, which may lead to strong alterations of population demographics and distribution [59,61,62]. *Myotis pilosus* is both a piscivore and an insectivore; when fish resources become limited, it will shift its diet to insects [18]. However, the activity of insects is affected by ambient temperature [63]. Low ambient temperatures reduce the activity of volant insects, and long-term low temperatures will delay the development of insects [64]. Thus, the temperature may influence the distribution of bats by acting on their food resources. Indeed, according to Park et al. (2000), *Rhinolphus ferrumequinum* increased its activity duration when the ambient temperature was higher than 10 °C, which is related to the thermal threshold of insect activity [65]. In addition, temperature may affect the hibernation and reproduction of bats. Many bats hibernate to survive harsh environments, such as low temperatures [66]. However, frequent hibernation of male bats would lead to sperm production delayed, which affects reproduction in the autumn [66]. For female bats, frequent hibernation leads to pregnancy prolongation and developmental delay of the fetal organs [67]. This is unfavorable as a short summer may not be enough for pups to learn how to fly and forage, and they may not be able to accumulate enough fat for migration or hibernation [63].

Climate-driven habitat shifts might influence the distribution of species. According to the IUCN Red List, the population of *M. pilosus* has been declining in recent years due to environmental degradation, habitat destruction, and other factors [19]. Our study predicts that the suitable habitat of *M. pilosus* will expand in the future. This may be because the temperature will increase under future warming scenarios and more areas will reach the temperature suitable for the survival of *M. pilosus* (Figure 4). A similar conclusion was drawn from the study of *Pipistrellus nathusii* in the UK where the areas suitable for *P. nathusii* were projected to triple by 2050, and the minimum temperature contributed most to the expansion [68]. However, when we considered the dispersal ability of *M. pilosus*, almost half of the predicted suitable habitats cannot be reached by 2050 and about 11% of predicted suitable habitats are unreachable by 2070 (Figure 5; Table 3). In addition, the total area of moderately and highly suitable habitats of *M. pilosus* under the current climate is likely to be just 40.56 × 10^4^ km^2^ (accounting for 4.23 % of China’s land area) or even smaller if we considered their dispersal ability (Table 3).

In the face of global climate change, organisms may respond to climate change by migrating to new regions, adapting to new environments, or going extinct [69]. Fortunately, there are no reports of bat extinction due to climate change [69]. Studies show that climate warming has caused many species to migrate and spread to high altitudes and latitudes [70,71]. Our results suggested that *M. pilosus* is likely to shift slightly to higher latitudes and altitudes in the future (Figure 4 and Figure 5; Table 3). The migration process predicted in our study seems to have occurred or is progressing, as Wu found that bats in China, including *M. pilosus*, mainly shifted northward in the past 50 years (1960s–2000s), and most of these changes were related to thermal indices [60]. The same trend was observed in *Lasiurus seminolus* in the United States [72] and *P*. *nathusii* in Europe [73]; both species expanded rapidly to high latitudes in response to climate change in recent decades. In recent years, there have been many studies on local adaptation. It has been reported that *R. ferrumequinum* in Italy may adapt to new environments by changing its body size (such as increasing forearm length) [74]. Another study has confirmed that bats can respond quickly to climate change by changing their phenology. Twenty-two years of monitoring at Bracken Cave showed that the spring migration and summer breeding cycles of bats were approximately two weeks ahead, which could be the bats’ responses to climate changes [75]. However, we still know little about whether there are phenotypic, genetic, or behavioral changes in *M. pilosus* associated with environmental adaptation.

China is the concentrated distribution area of *M. pilosus*, and the protection of *M. pilosus* in China is of great significance for its global conservation and persistence. According to the results of this study, several regions were identified as potential highly suitable habitats, such as southern Beijing, central and southern parts of Hainan, and other priority conservation areas (Figure 7). These areas are both the current distribution areas and probably the most suitable habitats for the survival of *M. pilosus* in the future, and should be given more attention when developing protection strategies. Our results showed that “TREE” is an important environmental factor affecting the distribution of *M. pilosus* (Table 2); thus, the contiguous forests around these areas should be protected. Although the predicted variables related to human disturbance, including “Light” and “human disturbance,” have relatively small effects on the distribution of *M. pilosus*, the anthropogenic impacts, such as night light, noise, and hunting, should still be restricted around these areas. In addition, given the particular foraging behavior of *M. pilosus*, we could conduct long-term monitoring of several *M. pilosus* populations and nearby freshwater resources in future studies, and integrate the transcriptomic and metabolomic technologies, to demonstrate the effects of freshwater quality on the growth, development, reproduction, and metabolism of *M. pilosus*.

## 5. Conclusions

Our results indicate that climate change might have an important influence on the distribution of *M. pilosus*. Temperature and precipitation are important environmental factors affecting its distribution. Minimum temperature of coldest month was predicted to be the most important environmental variable. With the increase in temperature in the future, the suitable habitats will probably expand and shift toward higher latitudes and altitudes, but the area of suitable habitats that *M. pilosus* could disperse and successfully colonize is likely to be reduced in 2050 and 2070. Five priority conservation areas in central and southern Hainan Province, southern Guangdong Province, central Guizhou Province, and southern Beijing were identified as potential future refugia. We suggest long-term monitoring of these areas, which could be important for the population persistence of *M. pilosus* and biodiversity conservation.

## Figures and Tables

**Table 1:** Environmental variables used in MaxEnt.

Category of Environmental Variables	Environmental Variables	Abbreviation
Climate	Annual Mean Temperature	Bio1
	Mean Diurnal Range	Bio2
	Isothermality	Bio3
	Temperature Seasonality	Bio4
	Maximum Temperature of Warmest Month	Bio5
	Minimum Temperature of Coldest Month	Bio6
	Temperature Annual Range	Bio7
	Mean Temperature of Wettest Quarter	Bio8
	Mean Temperature of Driest Quarter	Bio9
	Mean Temperature of Warmest Quarter	Bio10
	Mean Temperature of Coldest Quarter	Bio11
	Annual Precipitation	Bio12
	Precipitation of Wettest Month	Bio13
	Precipitation of Driest Month	Bio14
	Precipitation Seasonality	Bio15
	Precipitation of Wettest Quarter	Bio16
	Precipitation of Driest Quarter	Bio17
	Precipitation of Warmest Quarter	Bio18
	Precipitation of Coldest Quarter	Bio19
Land cover and vegetation	Land Use Type	Landuse
	Normalized Vegetation Index	NDVI
	Percent Tree Cover	TREE
Terrain	Altitude	
	Slope	
	Aspect	
Light index	Night Light Brightness	Light
Human disturbance	Human Influence Index	Hii
	Distance to Settlements	Dis_set
	Distance to Roads	Dis_roa
River	Distance to Freshwater	Dis_riv

**Table 2:** Dominant environmental variables and their contributions.

Environmental Variable	Percent Contribution (%)	Permutation Importance (%)
Bio6	25.9	52
Bio16	16.1	0.6
TREE	15.8	14.4
Bio14	14.6	0.9
Slope	7.1	10.9
Light	6.5	3.8
Landuse	6.2	1.3
Dis_riv	2.5	6.1
Bio15	2	4.2
Aspect	1.7	1.5
Dis_set	1.6	4.4

**Table 3:** Altitude and area of *M. pilosus* suitable habitats under current and future climate scenarios.

Years	Climate Scenario	Altitude (m)	Area (×10^4^ km^2^)				
		Average Altitude	Highly Suitable Habitats	Moderately Suitable Habitats	Lowly Suitable Habitats	Total Area	Suitable Habitats with Limited Dispersal
Current	-	648.19	5.74	34.82	119.98	160.54	-
2050	RCP2.6	681.94	6.87	39.15	123.55	169.58	94.00
	RCP4.5	698.58	6.92	40.71	123.86	171.48	95.00
	RCP8.5	692.79	7.49	38.00	124.25	169.74	94.14
2070	RCP2.6	678.94	6.97	41.15	123.35	171.47	154.16
	RCP4.5	687.75	7.67	41.14	124.86	173.67	155.16
	RCP8.5	708.56	9.70	41.43	127.87	179.00	156.99

**Table 4:** The area of climate refugia under different climate scenarios for *M. pilosus*.

Climate Scenario	In Situ Refugia Area/(×10^4^ km^2^)		Ex Situ Refugia Area/(×10^4^ km^2^)	
	2050	2070	2050	2070
RCP2.6	3.87	4.77	0.86	1.22
RCP4.5	3.80	4.76	0.92	1.74
RCP8.5	3.77	4.51	1.36	3.55

## Data Availability

The data from this study are available from the corresponding authors upon reasonable request.

## Supplementary Materials

The following supporting information can be downloaded at: https://www.mdpi.com/article/10.3390/ani13111784/s1, Table S1: *Myotis pilosus* occurrence coordinates in China; Table S2: Analysis of variable contributions for the current suitable habitats of *M. pilosus*; Figure S1: Correlation analysis based on 26 environmental variables; Figure S2: Distribution pattern of *M. pilosus* under current climate scenario; Figure S3: Distribution of in situ and ex situ refugia for *M. pilosus* under different climate scenarios.
