# Supplementary material for: Effects of Climate Change on the Distribution of Threatened Fishing Bat Myotis pilosus in China
Source: Animals (Basel). 2023 May 27;13(11):1784. doi: 10.3390/ani13111784 (PMC10251902; doi:10.3390/ani13111784)
Supplement: Supplementary file 1 [file animals-13-01784-s001.zip › animals-2340346-supplementary.pdf]

animals

MDPI

Table S1 *Myotis pilosus* occurrence coordinates in China

Species	Longitude/°	Latitude/°
Myotis pilosus	99.16	25.13
Myotis pilosus	100.55	25.49
Myotis pilosus	100.73	22.62
Myotis pilosus	102.34	24.50
Myotis pilosus	104.44	31.63
Myotis pilosus	105.69	25.44
Myotis pilosus	105.78	26.06
Myotis pilosus	107.15	28.23
Myotis pilosus	107.44	33.73
Myotis pilosus	107.57	29.17
Myotis pilosus	107.75	31.19
Myotis pilosus	109.89	18.85
Myotis pilosus	109.93	32.63
Myotis pilosus	110.27	20.01
Myotis pilosus	110.36	25.29
Myotis pilosus	110.95	19.65
Myotis pilosus	111.33	25.30
Myotis pilosus	111.42	33.30
Myotis pilosus	111.96	22.47
Myotis pilosus	114.12	23.65
Myotis pilosus	114.57	29.16
Myotis pilosus	115.73	27.23
Myotis pilosus	115.73	39.71
Myotis pilosus	116.55	31.47
Myotis pilosus	116.94	36.44
Myotis pilosus	117.40	40.05
Myotis pilosus	117.67	36.6
Myotis pilosus	117.69	28.21
Myotis pilosus	117.70	29.39
Myotis pilosus	117.94	27.63
Myotis pilosus	118.28	28.46
Myotis pilosus	120.03	30.12
Myotis pilosus	120.62	31.31

Table S2 Analysis of variable contributions for the current suitable habitats of *M. pilosus*.

Variable	Percent contribution	Permutation importance
Bio6	19.3	51.3
TREE	10.6	4.5
Slope	9.5	7
Bio12	8.9	0
Landuse	8.2	1.9
Bio14	8	0.2
Bio16	7.4	1.4
Light	7	3.6
Bio17	6.8	0
Bio18	2.7	1.1
Dis_riv	2	5.8
Bio15	1.8	5.4
Aspect	1.6	0.1
Dis_set	1.1	3.8
Bio1	0.9	1.5
Altitude	0.8	5.5
NDVI	0.6	3.3
Bio13	0.5	1.2
Bio19	0.5	0.3
Bio2	0.4	0.2
Bio4	0.4	0.1
Dis_roa	0.3	0.6
Hii	0.2	1.1
Bio9	0.2	0
Bio7	0	0
Bio8	0	0

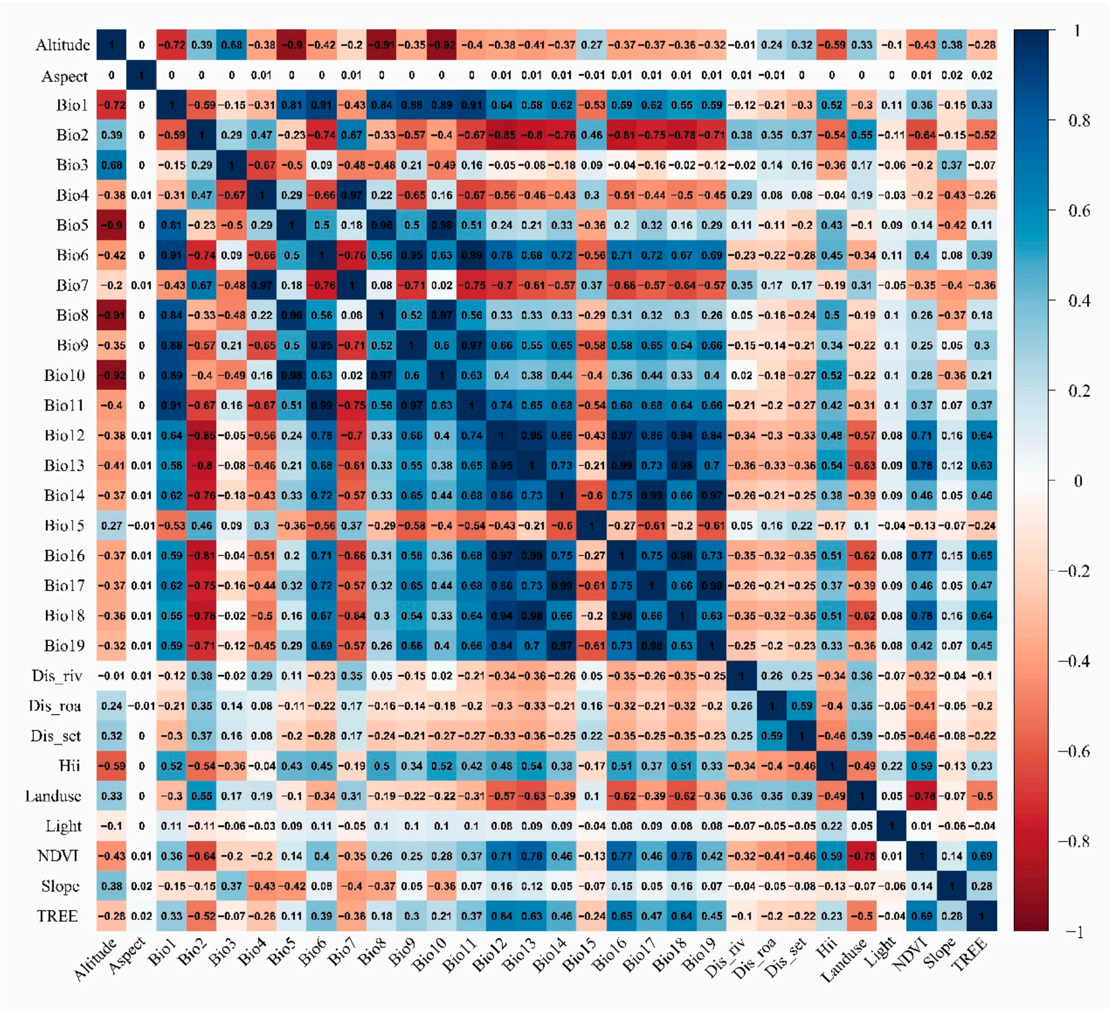

Figure S1 Correlation analysis based on 30 environmental variables.

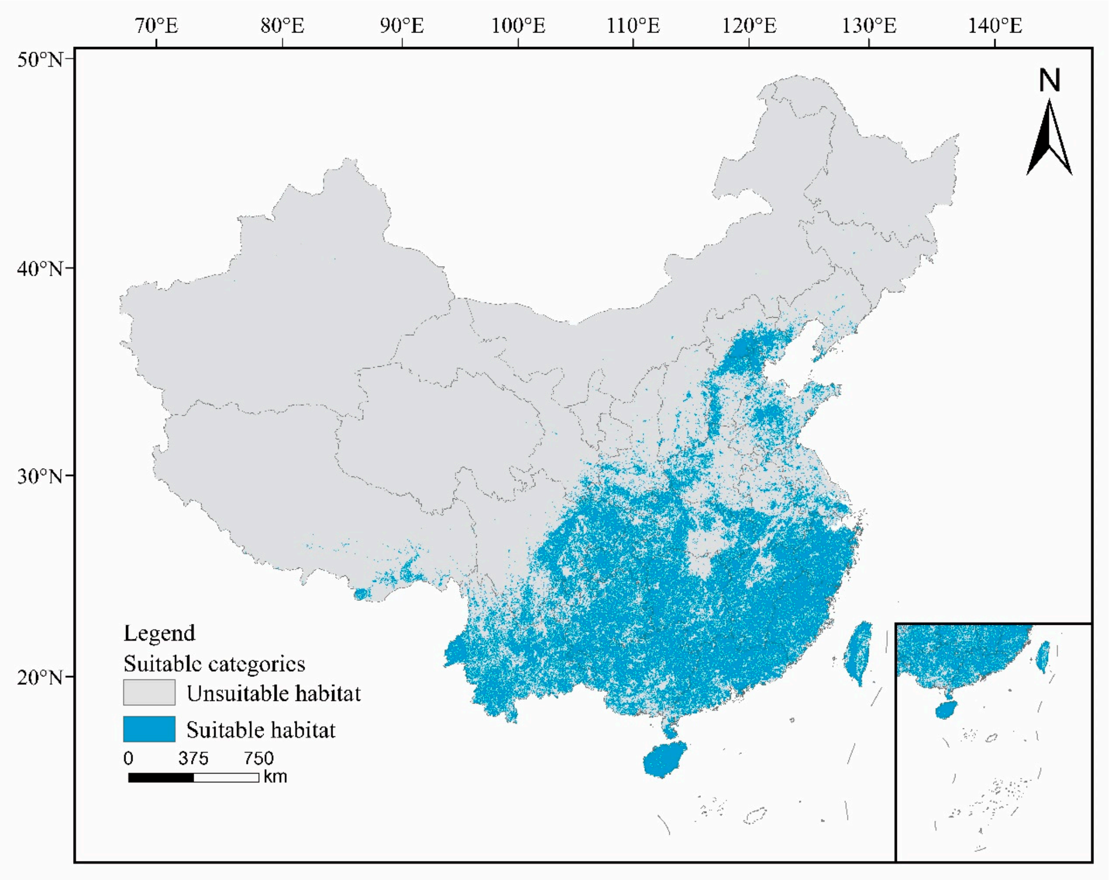

Figure S2 Distribution pattern of *M. pilosus* under current climate scenario.

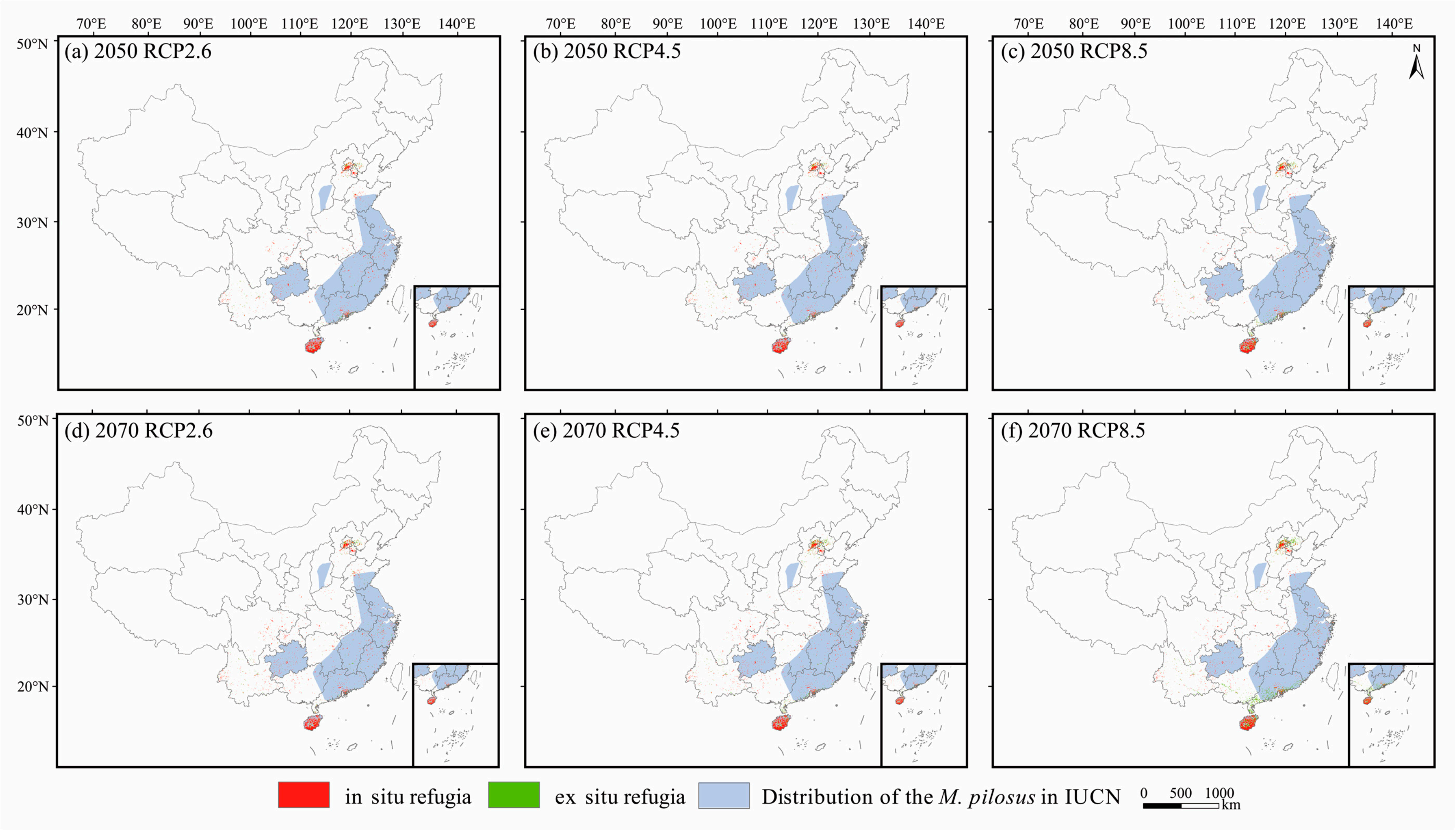

Figure S3 Distribution of in situ and ex situ refugia for *M. pilosus* under different climate scenarios.
